# Fat necrosis associated with glucagon-like peptide-1 receptor agonist- induced weight loss

**DOI:** 10.1016/j.jdcr.2026.08.033

**Published:** 2026-09-03

**Authors:** Fahd A. Malik, Jordan Tanner, M. Yadira Hurley, Emily Smith, Tyler Hooton

**Affiliations:** Department of Dermatology, Saint Louis University, St. Louis, Missouri

**Keywords:** adipose tissue remodeling, encapsulated fat necrosis, fat necrosis, GLP-1 receptor agonist related pain, GLP-1, incretin therapy, lipoatrophy, painful lumps, painful nodules, tirzepatide, weight loss, weight loss nodules

## Introduction

Painful subcutaneous adipose nodules are an uncommon dermatologic presentation with a broad differential diagnosis that includes adiposis dolorosa, panniculitis, lipomatosis, lipoatrophy, and fat necrosis. Fat necrosis is most often associated with trauma, surgery, or injection-related injury and involves a complex and not fully understood process. Fat necrosis generally involves cell necrosis or apoptosis that leads to the recruitment of fibroblasts and macrophages, eventually causing lipid-laden histocytes to accumulate between cyst-like areas.^1^ However, progression of healthy adipose to fat necrosis is complex as cell injury can occur via different mechanisms. Factors such as cytokines, ischemia, and heat can drive apoptosis and necrosis of an adipocyte.^2^ Associations between fat necrosis and individual patient factors such as smoking, older age, and obesity further supports the multi-faceted mechanistic potential for fat necrosis occurrence.^1^

Glucagon-like peptide-1 receptor agonists and dual incretin therapies are increasingly used for weight management and can induce rapid and substantial weight loss.^3^ As the use of these agents expands, recognition of potential adipose-related cutaneous adverse effects is important. We report a case of painful subcutaneous fat necrosis developing after rapid medication-associated weight loss in a patient treated with tirzepatide.

## Case report

A 73-year-old woman presented to the dermatology clinic with a 3-month history of progressively painful subcutaneous nodules on the extremities. She reported a preceding intentional weight loss of approximately 27 kg over the prior 6 months while using tirzepatide (Zepbound). The nodules appeared after this period of rapid weight loss, initially asymptomatic and subsequently becoming severely tender to palpation. She described the lesions as BB pellet–sized and symmetrically distributed over the bilateral upper arms and thighs. She denied antecedent trauma, injections, or localized injury.

Physical examination revealed numerous soft, mobile, tender subcutaneous nodules without overlying epidermal or dermal changes, most prominently involving the thighs. Individual nodules measured approximately 5-10 mm in diameter. Given the clinical findings, diagnostic options were reviewed and a biopsy was performed.

A 6 mm punch biopsy obtained from a palpable nodule on the left thigh demonstrated adipose tissue with fat necrosis. Histopathologic sections showed a relatively circumscribed collection of mature adipose tissue with focal aggregates of lipid-laden histiocytes (Figs 1 and 2). Features of lipoatrophy were also present (Fig 3).

**Fig 1:**
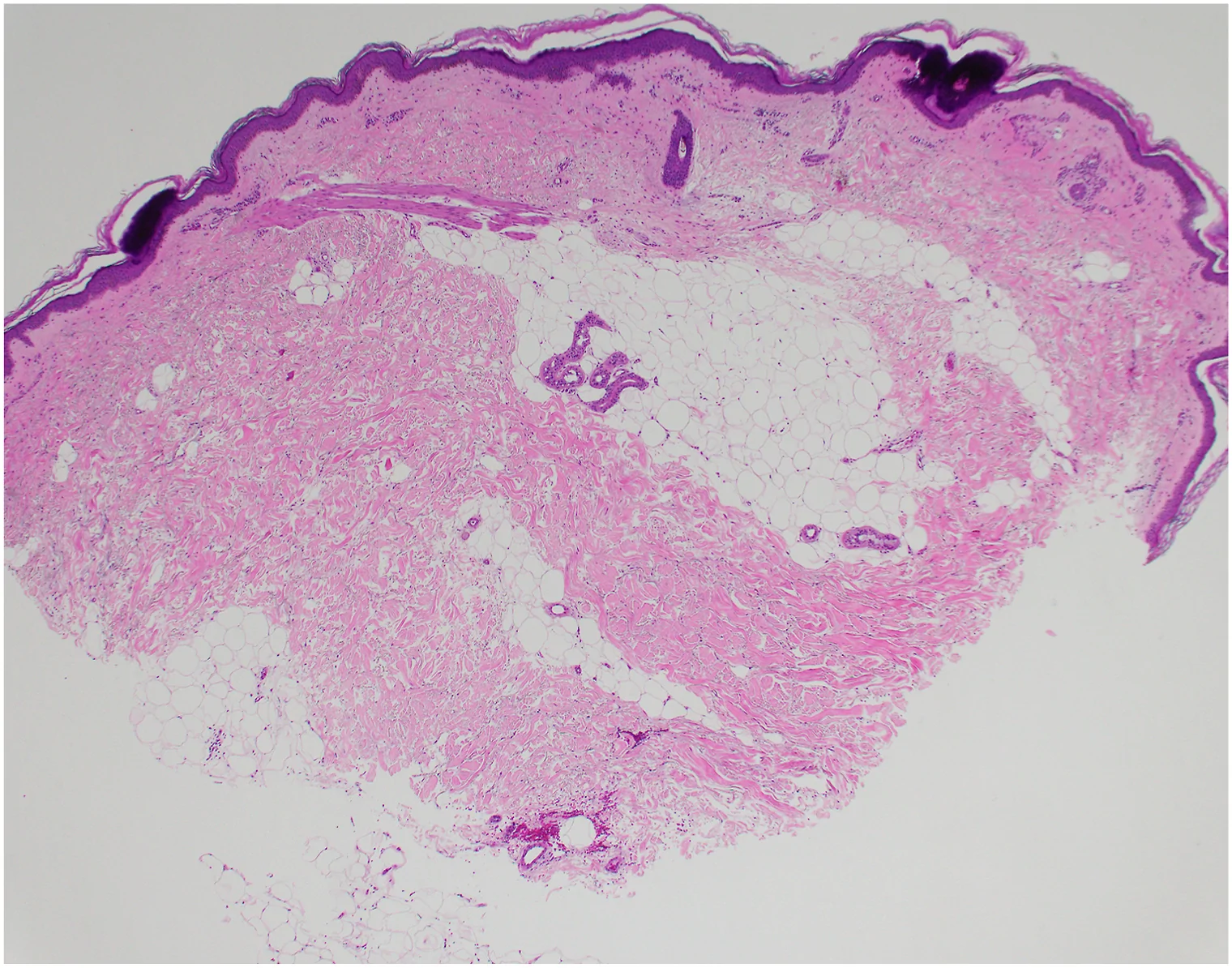
Histopathology from left thigh biopsy of subcutaneous fat necrosis associated with GLP-1 agonist-induced weight loss; Low power view, epidermis and dermis visible, mild variation in adipocyte sizes. *GLP-1*, Glucagon-like peptide-1.

**Fig 2:**
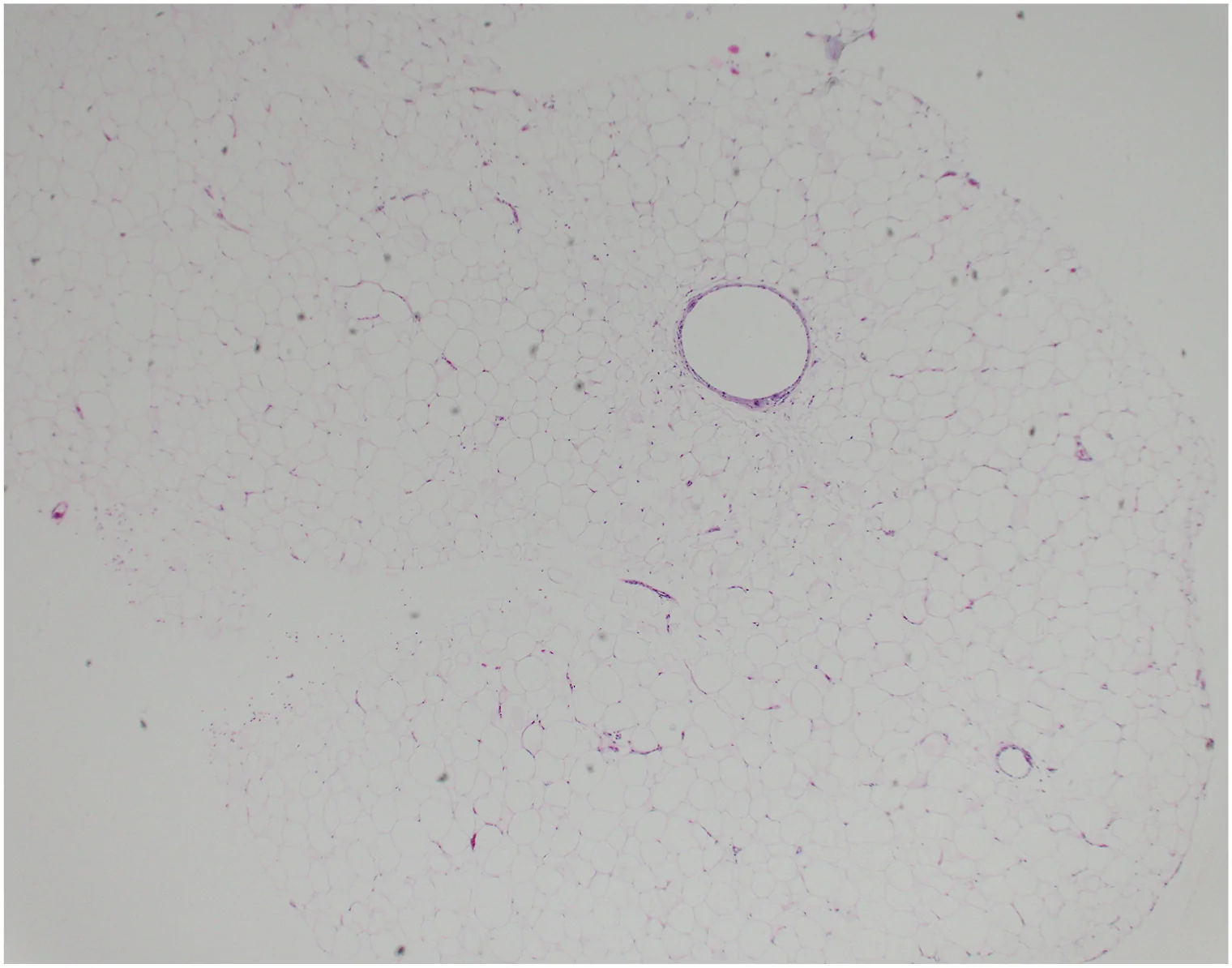
Histopathology from left thigh biopsy of subcutaneous fat necrosis associated with GLP-1 agonist-induced weight loss; Low power view, mild variation in adipocyte sizes, and focal pseudo-cystic aggregation of free lipid material consistent with fat necrosis. *GLP-1*, Glucagon-like peptide-1.

**Fig 3:**
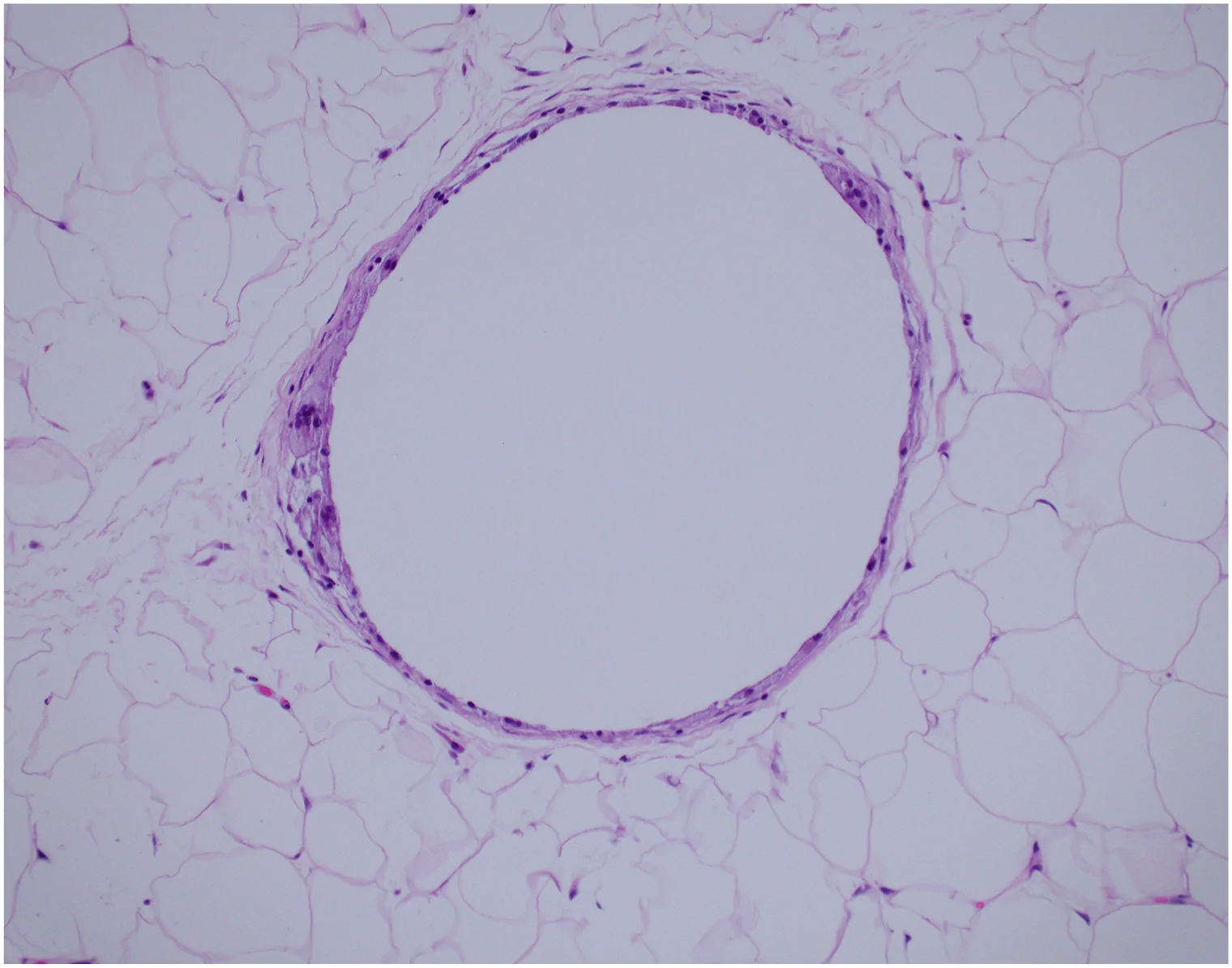
Histopathology from left thigh biopsy of subcutaneous fat necrosis associated with GLP-1 agonist-induced weight loss; High power view, pseudo-cystic aggregation of free lipid material with surrounding lipid-laden macrophages consistent with fat necrosis. *GLP-1*, Glucagon-like peptide-1.

At follow-up, the patient elected to discontinue tirzepatide due to additional adverse effects, including hair loss. Given the multiplicity and tenderness of the nodules, the differential diagnosis included adiposis dolorosa (Dercum’s disease) and other disseminated lipomatous disorders. However, given the histological findings of fat necrosis and timing of the occurrence of the nodules the findings were thought to be induced by rapid medication-associated weight loss. Lesions have not resolved 6 months following discontinuation of tirzepatide, although tenderness improved following discontinuation.

## Discussion

Painful subcutaneous adipose nodules encompass a broad differential diagnosis, including adiposis dolorosa (Dercum’s), lipomas, panniculitis, lipoatrophy, and local injection related fat necrosis. In this case, the patient’s symmetric distribution of tender nodules and diffuse involvement raised initial concern for a widespread lipomatous process such as adiposis dolorosa. However, the temporal relationship between significant weight loss and symptom onset, along with histopathologic findings of fat necrosis, suggested an alternative mechanism.

Similar in presentation, adiposis dolorosa is characterized by multiple painful lipomas, most commonly affecting middle-aged women and frequently associated with obesity, metabolic disease, and chronic pain syndromes.^4^ Histopathologic findings are often nonspecific and resemble benign lipomas, with pain hypothesized to arise from inflammatory, neuropathic, or microvascular mechanisms. Although the patient’s clinical presentation shared some features of adiposis dolorosa, the small size of the nodules, absence of discrete lipomatous masses, and presence of focal fat necrosis on biopsy were atypical.

The patient reported a substantial weight loss of approximately 27 kg over 6 months, preceding the onset of her painful nodules, in the setting of tirzepatide use. Rapid weight loss has been associated with adipose tissue remodeling, ischemia, and inflammatory change, which may predispose to fat necrosis.^5^ The histological findings of mature adipose tissue with lipidized histiocytes are consistent with this process. Features of lipoatrophy considered on pathology further support an evolving adipose disorder rather than a static condition, such as a lipoma.

Glucagon-like peptide-1 receptor agonists and dual incretin agonists, including tirzepatide, are increasingly used for weight management and can induce marked and rapid reductions in adipose volume.^3^ Sudden adipocyte shrinkage and altered vascular supply may result in localized hypoxia, inflammation, and subsequent fat necrosis.^5^ The diffuse and symmetric distribution of nodules in this case suggests a systemic adipose response rather than localized trauma or injection-related injury.

Management of painful adipose disorders is largely supportive and individualized. In this case, discontinuation of medication assisted with pain control.

This case highlights fat necrosis as a potential complication of rapid medication-associated weight loss and underscores the importance of considering systemic adipose changes in patients presenting with new-onset painful subcutaneous nodules. As the use of glucagon-like peptide-based therapies continues to expand, recognition of medication-associated adipose injury may allow for earlier diagnosis, avoidance of unnecessary procedures, and informed discussion regarding continuation or rate of weight loss therapy. Further studies are needed to definitively elucidate the pathophysiology and optimal management of weight loss-associated adipose necrosis.

## Conflicts of interest

None disclosure.
